# A novel filtration system based on ceramic silver-impregnated pot filter combined with adsorption processes to remove waterborne bacteria

**DOI:** 10.1038/s41598-020-68192-y

**Published:** 2020-07-08

**Authors:** Sandra Patricia Rivera-Sánchez, Iván Darío Ocampo-Ibáñez, Jorge Antonio Silva-Leal, Liliana Janeth Flórez-Elvira, Ana Valentina Castaño-Hincapié, Andreina Dávila-Estupiñan, Jorge Iván Martínez-Rivera, Andrea Pérez-Vidal

**Affiliations:** 1 Research Group of Microbiology, Industry and Environment, Faculty of Basic Sciences, Universidad Santiago de Cali, Calle 5 # 62-00, Cali, Valle del Cauca 760035 Colombia; 2 Research Group in Electronic, Industrial and Environmental Engineering, Faculty of Engineering, Universidad Santiago de Cali, Calle 5 # 62-00, Cali, 760035 Colombia; 3 Department of Public Health and Community Medicine, Universidad Icesi, Calle 18 # 122-135, Cali, Colombia

**Keywords:** Water microbiology, Environmental microbiology

## Abstract

Halving the proportion of the people without sustainable access to safe drinking water and basic sanitation is among the Sustainable Development Goals (SDG). Lack of access to safe drinking water has been associated with the prevalence of waterborne diseases. Due to this reported association, the development of household water treatment devices has been an alternative to improve the quality supply of domestic water. In this study, we aimed to evaluate the performance of a ceramic silver-impregnated pot filter (CSF) system coupled with an adsorption process, composed of silver-impregnated granular activated carbon and zeolite (CSF + GAC-Z), to remove waterborne bacteria *Escherichia coli* and *Salmonella* spp. from spiked water. The performance of this system was compared with the conventional CSF system. In this respect, we evaluated six CSF and six CSF + GAC-Z using spiked water with 10^3^ and 10^2^ CFU/mL of *E. coli* and *Salmonella* spp. The mean percentage of removals ranged between 98% and 99.98%. The highest bacterial removal efficiency was recorded by the CSF + GAC-Z (99%) and CSF (99.98%) for *E. coli* and *Salmonella* spp., respectively, but no significant statistical differences were found between filtration systems. Our findings suggest that the CSF + GAC-Z system was effective in the removal of waterborne bacteria from spiked water.

## Introduction

According to World Health Organization (WHO), the access to safe drinking water is essential for health protection and sustenance of life, because this is required for all usual domestic purposes, including drinking, food preparation and personal hygiene^1^. However, about 1.8 billion people around the world use water polluted by faecal material which includes pathogenic microorganisms, such as *Escherichia coli* and *Salmonella* species (*Salmonella* spp.)^2–6^. These enteric pathogens are causative agents of waterborne diseases, and they can be transmitted to humans by ingestion of contaminated water, creating serious complications, including diarrhea and even death^5,7,8^. About 2.2 million deaths globally are associated with diarrhea each year, because of the reported association between microbiological contamination of drinking water and the prevalence of waterborne diseases ^2,3,9–11^. This situation is striking in most developing countries where access to basic sanitation services and safe drinking water is very limited^2–4,12,13^. About 38 million people do not have access to sources of drinking water in Latin America and the Caribbean, and the water-related diseases are among the ten principal causes of death every year in this region^14^. In Colombia, the drinking water reached a coverage of 97% at the national level in 2015, but the quality of water varies according to its source as well as its location^13–15^. In this respect, the drinking water quality standards are not met in a high percentage of municipalities in Colombia, where problems related to the presence of *E. coli* and *Salmonella* spp. have been identified in urban and rural areas^15,16^.

Goal 6 included in the Sustainable Development Goals (SDG) is halving the proportion of the people without sustainable access to safe drinking water and basic sanitation by 2030^17^. In this respect, the lack of access to safe drinking water is a notable hindrance to the improvement in human health and development of the community in rural/urban areas in developing countries^13^. To counter this, several low-cost water treating methods at the household level have been developed and implemented to provide consistent access to safe drinking water in developing countries^12,18–21^. In this context, currently, the point-of-use water treatment (PoUWT) systems have shown to be a promising option for improving the water quality at the household level in rural areas of these countries^12,19,22–26^.

Several PoUWT systems can be used to provide microbiological, physical and chemical water treatment, including among others disinfection, particle filtration (for instance ceramic pot filters—CPF), adsorption media (for instance granular activated carbon—GAC) and combined systems^1,27–29^. The CPFs are commonly manufactured by combining clay, water, and sawdust to make a pot-shaped ceramic filter with small pores inside a plastic bucket. These CPFs physically remove colloidal particles and microorganisms, such as bacteria and even viruses, when water is passed through the filter and then the treated water is stored in the plastic bucket ^12,18–20,23,25,30–35^. These ceramic water filter systems are often impregnated with colloidal silver for disinfection purposes and control bacterial growth^19,20,22,23,30–32,36,37^. CPFs have been widely evaluated for efficient and effective removal of bacteria over the short term, showing significant reductions of pathogenic bacteria concentration in raw and spiked waters after the filtration^12,18–20,22,23,25,31–33,36^. On the other hand, the activated carbon and zeolite no impregnated with colloidal silver have shown an important microbicidal property, in order to reduce the risk of water contamination with microorganisms^1,38–44^. GAC and activated carbon fibers (ACF) are generally impregnated with silver to enhance their antibacterial effect, which has demonstrated a strong bactericidal effect against *E. coli*^38,39^*.* GAC can be integrated into common filtration methods to improve the efficiency and effectiveness of the filter systems, in order to produce water of acceptable drinking quality^37,42^.

In this study, we evaluated the performance of a modified point-of-use ceramic silver-impregnated pot filter (CSF) system coupled with an adsorption process, composed of silver-impregnated granular activated carbon (GAC) and zeolite (CSF + GAC-Z) to remove *E. coli* and *Salmonella* spp. The performance of this modified system was compared with a conventional CSF system. This is the first evaluation of a CSF system coupled with an adsorption process to remove waterborne bacteria from spiked water. The two enteric bacteria were included in this study because they are commonly used as an index of pollution in water globally, the water is known to be a common vehicle for their transmission, and they are related to waterborne intestinal diseases outbreaks in developing countries^1,6–8,33,45^.

## Materials and methods

### Filters characteristics

**Figure 1:**
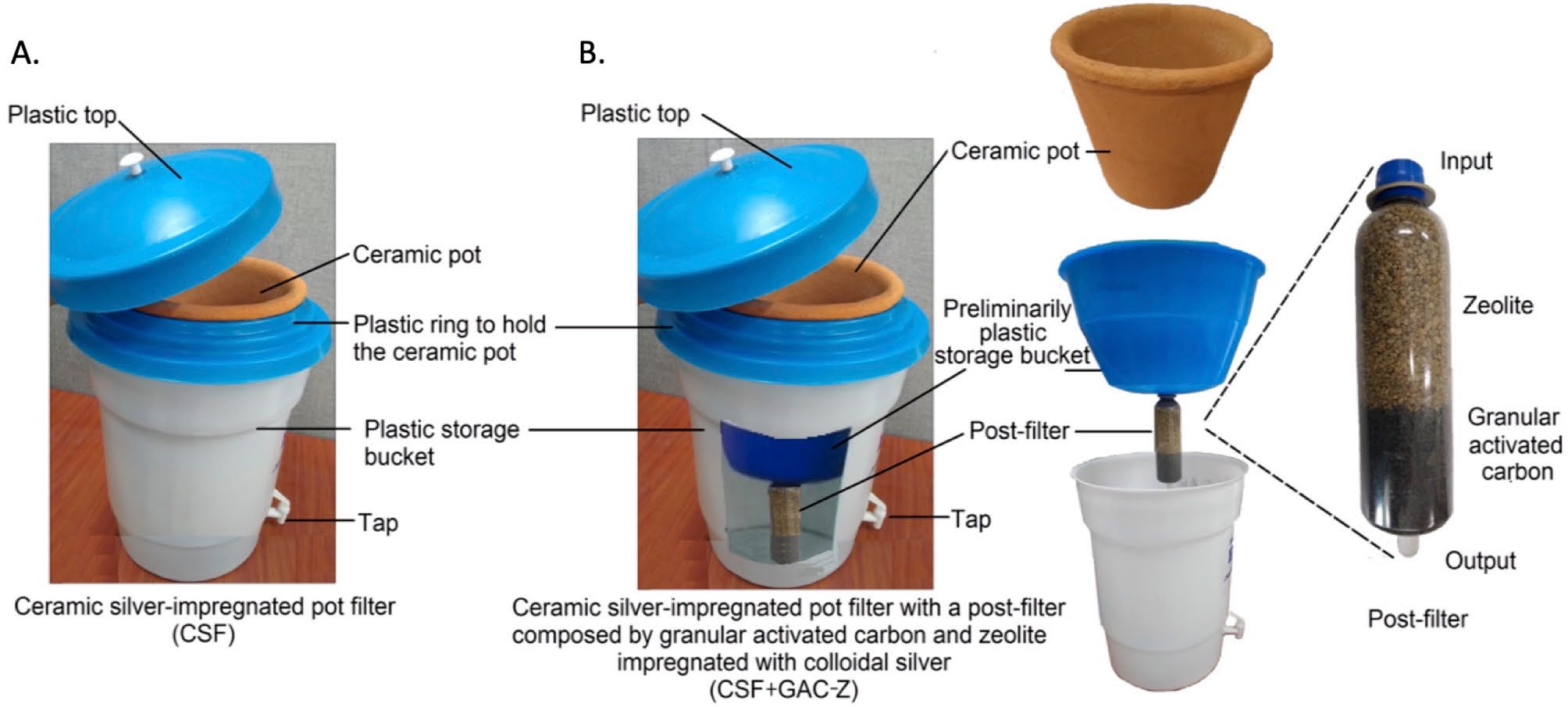
Ceramic-based point-of-use water treatment systems. (**A**) Ceramic silver-impregnated pot filter (CSF). (**B**) Ceramic silver-impregnated pot filter with a post-filter composed of granular activated carbon (GAC) impregnated with colloidal silver and zeolite (Z) (CSF + GAC-Z).

Two types of point-of-use CPFs were evaluated in this study. The CSF is made of red clay and coconut husk impregnated with colloidal silver in a 500 ppm concentration. The pot-shaped ceramic filter is located inside a 25-L plastic bucket which serves as a filtered water storage container (Fig. 1A). The CSF + GAC-Z filter is a CSF modified. This modified system includes a preliminarily plastic storage bucket, which receives the water initially filtered by the pot-shaped ceramic filter, and a post-filter made of granular activated carbon impregnated with colloidal silver and zeolite. This system is located inside a 25-L plastic bucket which serves as the last storage container of filtered water (Fig. 1B).

### Preparation of spiked water

To evaluate the performance of the filters, tap water spiked with *E. coli* and *Salmonella* spp. was prepared. Firstly, sodium thiosulfate pentahydrate (Na_2_S_2_O_3_.5H_2_O) in a 20 mg/L concentration was added to eliminate the free residual chlorine of tap water. A color disc test kit (HACH, CNe70F) was used to verify the absence of chlorine and avoid an excess of thiosulfate. Before the addition of bacteria to tap water, kaolin (0.12 g/L) was added to obtain a value near to 30 Nephelometric Turbidity Unit (NTU), as well as analytical grade NaCl (Merck, 0.12 g/L) to obtain a concentration of 100 mg/L of Total Dissolved Solids (TDS) to avoid the early lysis of bacterial cells^46–48^. Then, the tap water was spiked with *E. coli* and *Salmonella* spp. to concentrations of 10^3^ CFU/mL (spiked water 1) and 10^2^ CFU/mL (spiked water 2). In order to do this, *E. coli* (ATCC 25922) and *Salmonella* spp. (ATCC 14028) strains were used. These strains were obtained from the American Type Culture (Collection Rockville, MD.T) and their purity was verified with crystal biochemical tests for Enterobacteriaceae non-fermenters of BBL-BD. In order to prepare spiked water, first, a bacterial growth calibration graph according to the McFarland scale between 0.5 and 10 at 620 nm wavelength was made (Thermo Scientific Genesys 20 Spectrophotometer). Then, the preparation of the inoculum was started by reactivating the strains, transferring 20 mL to 150 mL of nutrient broth (Merck, 105443) followed by an incubation period between 18 and 24 h and 35 ± 2 °C, to obtain the bacteria growth stationary phase. The cell washing procedure and the initial bacteria adjustment were carried out following the methodology of Rivera et al*.*^49^. Additionally, the microbiological quality of tap water used to prepare spiked water was periodically verified in terms of the heterotrophic bacteria, *Pseudomonas*, Total Coliforms, *E. coli,* and *Salmonella* spp. according to the standard methods^50^. Finally, as a result of the bacterial replication time being 30 min and the filtration time was three hours, the bacterial regrowth during filtration time was controlled. In order to do this, the bacterial concentration was measured in a surplus of non-filtered spiked water immediately after inoculation and again after three hours. The bacterial concentration after three hours was considered as the initial bacterial concentration of the spiked water before filtration.

### Performance evaluation of the filtration systems

All experiments were carried out in the Universidad Santiago de Cali located at an altitude of 1018 m above the sea. A total of 12 new filters were used, six CSF and six CSF + GAC-Z, for the long-term performance study. The filtration systems were operated in batch mode at an average environmental temperature of 21 °C for 135 days, changing the microbiological quality of the spiked water every five weeks. In order to reach an average microbiological removal efficiency of filters, a progressive periodic reduction of the bacteria concentration was made using spiked water, starting the operation of systems with *E. coli* and *Salmonella* spp. concentration of 10^3^ CFU/mL, then it was reduced to 10^2^ CFU/mL.

A total of 50 L of spiked water was prepared daily for CSF and CSF + GAC-Z systems and 7.5 L was added to each filter. The total amount of filtered water used during the evaluation was 1013 L per filter. The sampling of the filtered water was made three hours after the filter was full. The ceramic pot and the plastic parts of the filtration systems were cleaned daily with tap water and a soft brush. Two times per month we measured total silver in the filtered effluents to control the silver leaching (n = 10)^50^. When the filtration rate efficiency decreased, the systems were cleaned using a sponge and water with a sodium hypochlorite concentration of 4%, and the remaining chlorine content after cleaning was reduced by washing the filters with sufficient tap water. The evaluation of filtration systems was carried out determining the bacteria concentrations for spiked water as well as for the filtered water through membrane filtration—SM9222B for *E. coli* and Most Probable Number (MPN) —SM9225C3 for *Salmonella* spp.^49,50^.

### Statistical analysis

The results were analyzed using descriptive statistical tools with median and interquartile range. The removal efficiencies of the systems were evaluated using the Kruskal–Wallis test, a non-parametric alternative to the ANOVA test, with the R-Project free software Version 1.1.463. The average log reduction value (LRV) was calculated using colony-forming units (CFU) and most probable number (MPN) values for every individual sample.

## Results and discussion

### Analysis of spiked water

The microbiological characteristics of the spiked water initially prepared to evaluate filtration systems in this study were consistent with those suggested by EPA^46^ with a maximum critical value of 10^4^ CFU/mL and 30 NTU (Table 1). The water turbidity average was 29.3 ± 5.6 NTU. However, an increase in the bacterial concentration was found in the spiked water after three hours. The log increase of *E. coli* was 0.2, whereas an increase of *Salmonella* ranged between 2.0 and 2.7 logs was observed (Table 1). This high increase observed in *Salmonella* concentration can be explained by the high sensitivity of the MPN in comparison to membrane filtration for bacterial detection in water^51,52^. The membrane filtration has shown to be less sensitive than fermentation tube techniques, which can detect small numbers of bacteria in waters^51,52^. In this respect, a log reduction of 0.1 for *E. coli* was found in the surplus of spiked water 1 after three hours, suggesting a sub-estimation related to the detection method used for this bacterium (Table 1).

**Table 1:** Microbiological characteristics of spiked water.

Spiked water	Bacteria	Units	n	Initial concentration^a^	Final concentration^b^
1	*E. coli*	CFU/100 mL	5	1.2 × 10^3^	0.8 × 10^3^
	*Salmonella* spp.	MPN/mL	5	2.4 × 10^3^	2.3 × 10^5^
2	*E. coli*	CFU/100 mL	5	1.6 × 10^2^	3.0 × 10^2^
	*Salmonella* spp.	MPN/mL	5	5.0 × 10^2^	2.3 × 10^5^

^a^Bacterial concentration of spiked water immediately after inoculation.

^b^Bacterial concentration in the surplus of spiked water after three hours.

### Performance of the filtration systems for the removal of waterborne bacteria

The total silver concentration in filtered effluents was lower than ranged 0.01 mg/L. These values were lower than levels of silver in drinking-water recommended by EPA^46^ and WHO^13^ to protect people from the possible health effects from long-term exposure to silver^46^. Additionally, this result suggests the low mobility of colloidal silver.

Both filter systems evaluated in this study were able to decrease the concentration of bacteria in spiked water samples (Tables 2 and 3). However, the bacterial removal had some variations depending on the performance of the filters and target microorganisms. The removal reached in this study ranged between 1.8 and 3.6 logs for both filter systems here evaluated (Tables 2 and 3). Overall, the LRVs found in this study are similar to those found in other studies where bacterial removals by silver-impregnated porous pot filters have been reported to range between 1 and 3.5 log_10_ reductions^20,21,31,32,47,53^.

**Table 2:** Concentrations of *E. coli* in the spiked water before and after filtration.

Spiked water	n	Filtration system	CFU/100 mL			Removal		*p* value
			Concentration before filtration^a^	Concentration after filtration^a^	IQR	LRV (average)	Efficiency (%)	
1	30	CSF	0.87 × 10^3^	3.5 × 10^1^	5 × 10^0^–5.9 × 10^1^	1.8	98.0	0.25^NS^
	30	CSF + GAC-Z	0.87 × 10^3^	1.6 × 10^1^	0–4.1 × 10^1^	2.0	99.0	
2	30	CSF	3.00 × 10^2^	4.0 × 10^0^	0–7.0 × 10^0^	2.0	99.0	0.67^NS^
	30	CSF + GAC-Z	3.00 × 10^2^	3.0 × 10^0^	0–7.0 × 10^0^	2.0	99.0	

*CFU* Colony-forming unit, *IQR* Interquartile range, *LRV* Log Reduction Value.

^a^Median.

NS, Non-significant differences found with a significance level of 0.05.

**Table 3:** Concentrations of *Salmonella* spp. in the spiked water before and after filtration.

Spiked water	n	Filtration system	MPN/100 mL			Removal		*p* value
			Concentration before filtration^a^	Concentration after filtration^a^	IQR	LRV (average)	Efficiency (%)	
1	30	CSF	2.30 × 10^5^	2.0 × 10^0^	2.0 × 10^0^–2.3 × 10^3^	3.4	99.96	0.68^NS^
	30	CSF + GAC-Z	2.30 × 10^5^	2.3 × 10^3^	2.0 × 10^0^–2.3 × 10^3^	3.2	99.94	
2	30	CSF	2.30 × 10^5^	1.2 × 10^2^	0.3 × 10^2^–2.3 × 10^2^	3.6	99.98	0.66^NS^
	30	CSF + GAC-Z	2.30 × 10^5^	0.8 × 10^2^	0.4 × 10^2^–7.0 × 10^2^	3.5	99.97	

*MPN* Most Probable Number, *IQR* Interquartile range, *LRV* Log Reduction Value.

^a^Median.

NS, Non-significant differences found with a significance level of 0.05.

The percentage removal of *E. coli* and *Salmonella* spp. from spiked water by the CSF ranged between 98% and 99.98%, which were within percentages reported in previous studies where the removal rates ranged between 84 and 100%^21,25,31,53^. In this respect, the log_10_ reductions of 1.8 for *E. coli* (Table 2) were slightly lower in comparison to those found in most studies for this bacterial species (log_10_ reduction ranged between 2 and 3) using silver-impregnated pot filters^21,22,25,31,53^. On the other hand, removals of *Salmonella* spp. by silver-impregnated pot filters have been reported to range between 2 and 3 log reductions^25,31^, however, we found slightly higher removal values in this study with log_10_ reductions that ranged between 3.4 and 3.6 for CSF system (Table 3).

This is the first evaluation of the CSF + GAC-Z system, which includes a post-filtration device composed of silver-impregnated GAC and zeolite downstream of the silver-impregnated pot filter (Fig. 1). The log reduction of bacteria for this system ranged between 2 (99%) and 3.5 (99.97%) logs (Tables 1 and 2). Despite this CSF + GAC-Z system being never evaluated before, the filtration rates efficiency found here (Tables 1 and 2) were comparable to those reported in a study where GAC was integrated into ceramic filters (average removal of 99%) ^42^. However, we found a higher percentage of removal compared to GAC based-filters which showed poor performance with 80% of bacterial removal^54^.

When both systems were compared, no significant statistical differences were found between filtration systems for removal of both *E. coli* and *Salmonella* (*p* value > 0.05) (Tables 2 and 3), but the CSF device exhibited higher performance to remove *Salmonella* spp. (Table 3). However, it is recognized that adsorption processes are used for the removal of chemical contaminants such as heavy metals and organic substances^1^. In this respect, in future research, it is advisable to evaluate the microbiological and chemical risk of the CSF + GAC-Z system in an integrated way. This finding suggests that the silver-impregnated GAC and zeolite did not contribute to increasing the removal and disinfection efficiency of filters, despite the anti-microbial properties attributed to silver supported by Zeolite^41^ and activated carbon^38,39^. In contrast, the removal effectivity and efficiency of the CSF unit can be attributed to the silver-coating onto the pot before filtering, because of the bactericidal properties of silver and its uses as a disinfectant^22,31^. According to the results found in this study, an improvement of drinking water quality is obtained using CSF and CSF + GAC-Z systems. However, the filtered water is not adjusted to permissible limits of bacteriological parameters for drinking water according to WHO and Colombia standards, which establish a maximum value of 0 CFU/100 mL for *E. coli* in acceptable drinking water for human consumption^1,55^. Nevertheless, these systems were not evaluated using raw environmental water samples with lower concentrations of target potential pathogenic bacteria. When these types of water samples were used previously with CPFs, these systems showed performances of 100% for the removal of presumptive bacteria species^31^. In this context, this suggests that the operational limit of filter systems here evaluated is bottom than 1 × 10^3^ CFU/100 mL. Alternatively, an additional simple disinfection step of the filtered water could be included in CSF and CSF + GAC-Z systems. A combination of the filter with a silver-embedded ceramic tablet placed in the lower of the plastic bucket has shown to be an effective alternative to remove *E. coli* at 100% over the time, as a result of this method provides continual disinfection of filtered water^19^.

### Economic analysis and costing comparison of removal systems

The combination of CSF and adsorption process increases the basic cost of the system. In this context, a 31% increase in the cost of CSF + GAC-Z system compared to basic cost of the CSF was calculated. The cost of conventional CSF system was calculated to be COP$180,000 (US$53.1), whereas the cost of CSF + GAC-Z was COP$260,000 (US$76.62), when the adsorption system was included. Because of no significant differences were found in the bacterial removal efficiency between the CSF + GAC-Z and CSF systems, the proportions of communities who would be willing to pay for the increase of CSF + GAC-Z system cost would be lower. However, inclusion of adsorption system could be considered as an added value of CSF + GAC-Z, because of adsorption processes could remove of heavy metals and organic substances^1^, even though studies to evaluate the removal of chemical contaminants by CSF + GAC-Z system are necessary.

## Conclusion

The outcomes of this study showed that the CSF + GAC-Z system decreased the concentration of bacteria from spiked water. No significant differences were found between the CSF + GAC-Z filtration system and the conventional CSF system for the removal of pathogenic bacteria here compared. In this respect, the integration of GAC did not improve the efficiency and effectiveness of the CPF system. Regardless, high removal efficiencies of waterborne bacteria were observed with maximum log reductions of 3.6 log for *Salmonella* spp. and 2.0 log for *E. coli* with CSF and CSF + GAC-Z, respectively. Even though the bacterial removal efficiency was not 100% in spiked water, bacteriologically the water obtained after filtration can be categorized as 'low risk' as per WHO standards. Although no significant differences in the performance of bacterial removal between both systems were observed, the combination of CSF with adsorption process would be socially effective and efficient for the removal of chemical pollutants, which would be needed to be clarified through further research before its practical application.
